# Multilevel barriers and facilitators to behavioral health treatment among Latino sexual minority men

**DOI:** 10.1371/journal.pmen.0000153

**Published:** 2025-04-21

**Authors:** Alyssa Lozano, Nequiel Reyes, Dalton Scott, Daniel J. Feaster, Audrey Harkness

**Affiliations:** 1 School of Nursing and Health Studies, University of Miami, Coral Gables, Florida, United States of America; 2 Department of Public Health Sciences, University of Miami Miller School of Medicine, Miami, Florida, United States of America; UCL: University College London, UNITED KINGDOM OF GREAT BRITAIN AND NORTHERN IRELAND

## Abstract

Latino sexual minority men (Latino/x or Hispanic gay, bisexual, or men who have sex with men; LSMM) are less likely to use behavioral health treatment than non-Latino white adults. However, there are no measures that evaluate barriers or facilitators to behavioral health treatment for LSMM. The purpose of this study was to (1) evaluate the factor structure of a measure of multilevel barriers and facilitators to behavioral health treatment among LSMM and (2) examine the association between identified factors of barriers and facilitators and engagement in behavioral health treatment. We developed the Multilevel Barriers and Facilitators to Behavioral Health Treatment measure. We first examined the hypothesized factor structure of the Multilevel Barriers and Facilitators to Behavioral Health Treatment measure using confirmatory factor analyses (CFA) in a sample of 235 LSMM. Next, we evaluated the correlation between identified barrier and facilitator factors and the association of the factors with engagement in behavioral health treatment. The CFA included seven multilevel barriers and four multilevel facilitators. Language and immigration barriers were significantly associated with less engagement in behavioral health. Positive provider demeanor was associated with higher engagement. The Multilevel Barriers and Facilitators to Behavioral Health Treatment Measure captures structural factors (e.g., insurance issues), interpersonal factors (e.g., negative provider demeanor), and individual factors (e.g., knowledge) that could impact treatment engagement. This multilevel measure of barriers and facilitators can inform behavioral health researchers of which modifiable determinants to focus on to improve engagement in behavioral health treatment for LSMM.

## Introduction

Approximately 20% of adolescents and adults in the United States (U.S.) are affected by negative behavioral health outcomes [1]. Behavioral health refers to an individual’s mental and emotional health/well-being as well as substance use disorders [1]. Studies suggest that, compared to their heterosexual peers, sexual minorities are at higher risk for depression and psychological distress [2,3] and that having a sexual minority identity was associated with increased rates of substance use and substance use dependence disorders [4]. These disparities have been linked to the additional stressors sexual minorities experience due to stigma, discrimination [5,6]. Due to intersecting systems of stigma and discrimination linked to sexual and racial/ethnic minoritization, Latino sexual minority men (LSMM) are additionally likely to experience higher levels of minority stress compared to White sexual minority men [7] that may subsequently lead to poorer behavioral health [8]. This difference in minority stress between LSMM and White sexual minority men may be attributed to LSMM facing discrimination not only by their Latino family and peers, but also from their White sexual minority and non-sexual minority counterparts [9]. In a study of 224 white and Latino young adults who self-identified as lesbian, gay, and bisexual, researchers reported that higher levels of family rejection were associated with poorer health outcomes and the LSMM in this study reported experiencing the highest number of negative family reactions to their sexual orientation during their adolescence [10]. Additionally, in a qualitative study of 27 LSMM, many participants shared that they felt disconnected from their culture because of their sexual orientation and they reported discrimination from the gay White community [9].

One potential reason for behavioral health disparities among LSMM may be that Latinos’ cultural norms are often in conflict with the U.S.’s healthcare systems. For example, some Latinos believe that diseases may be caused by spiritual forces (i.e., the evil eye) or experiencing an imbalance of hot and cold as opposed to believe in the biomedical model of illness in medicine [11]. Latinos also come from an allocentric culture, meaning that there is more emphasis placed on group harmony than the individual which is why family is extremely important to Latinos [9]. Thus, for Latinos, the whole family is the patient and it is important for providers to always respect family hierarchies and establish a connection with immediate and extended family members [11]. Additionally, Latinos also have particular views about seeking services for behavioral health concerns. For instance, in a study of 30 Latino receiving outpatient treatment for depression, participants shared that in the Latino culture, there is an emphasis to be resilient and cope with life’s problems without needing antidepressants [12]. Latino men, in particular, may be uniquely impacted by their gender and ethnic identity as their experiences with depression and access to/engagement with mental health services are shaped by their personal relationship with their masculinity as well as how providers perceive Latino men [13].

Behavioral health disparities affecting LSMM are related to several multilevel factors that cause healthcare disparities among LSMM (i.e., low rates of behavioral health treatment relative to White SMM; [14,15]). For instance, discrimination in healthcare, language barriers, and documentation status are a few examples of barriers that contribute to healthcare disparities for LSMM [15]. Because LSMM face distinct, compounding stressors (e.g., language barriers, immigration-related issues, and acculturation [16]), LSMM have a unique cultural experience which can derail help-seeking behaviors. In a study of barriers to seeking specialty treatment for substance use disorder treatment among White, Black, and Latino adults, Latinos were the only racial/ethnic group to identify cultural factors (e.g., treatment services not being culturally tailored, treatment not being culturally accepted, providers not being able to relate to Latino patients) as a barrier for not seeking help for a substance use disorder [17]. Additionally, prior studies have suggested that even if Spanish language services are provided, Latinos may still feel discouraged from utilizing health services that are not perceived as being culturally appropriate and relevant [16,18]

Despite the barriers that may impede LSMM from seeking out behavioral health treatment, facilitators to enable LSMM’s access to behavioral health treatment do exist. In a qualitative study that assessed barriers and facilitators to seeking depression treatment among Latinos, participants indicated having a social partnership with their provider and providers being aware of cultural differences between themselves and their Latino patients were key facilitators [19]. Additionally, in a study of older Latino adults, researchers found that those who received support and encouragement from their primary care provider and their family were more likely to utilize mental health services [20].

It is evident that there are various determinants that may enable or impede LSMM’s engagement in behavioral health treatment. However, to our knowledge, there are no measures that quantitatively assess these multilevel determinants of behavioral health treatment use among LSMM. A lack of multilevel measures of behavioral health treatment determinants among LSMM could impede fairness in the amount of scientific progress toward documenting and addressing multilevel determinants (i.e., barriers and facilitators) of behavioral health treatment engagement among LSMM [21]. For instance, a quantitative measure of barriers and facilitators to behavioral health treatment, could facilitate research examining how these determinants may impact behavioral health engagement.

A prior study conducted by our team addressed the gap in available measures by developing the Multilevel Barriers and Facilitators to Behavioral Health Treatment measure. Through qualitative interviews with LSMM and community stakeholders, we identified multilevel barriers and facilitators to accessing behavioral health services for LSMM that helped inform the development of our quantitative measure of barriers and facilitators to behavioral health [22]. The measure is multilevel, such that it examines barriers and facilitators at the individual (e.g., behavioral health treatment knowledge), interpersonal (e.g., behavioral health treatment navigation support), and cultural/structural (e.g., language/immigration concerns) levels, allowing for a broad assessment of determinants that may impact use of behavioral health treatments among LSMM.

This measure, however, has not yet been validated and little research has examined how this measure may relate to engagement in behavioral health treatment. Therefore, the purpose of this study is to (1) evaluate the factor structure of a measure of barriers and facilitators to behavioral health among a group of LSMM in South Florida, (2) examine the extent to which identified barrier and facilitator factors are related to each other (i.e., discriminant validity), and (3) examine the extent to which identified barrier and facilitator factors are related to behavioral health treatment engagement (i.e., convergent validity). We hypothesize that the Multilevel Barriers and Facilitators to Behavioral Health Measure will have two dimensions: barriers and facilitators within a priori (i.e., based on qualitative findings) theoretically grouped domains. We also hypothesize that behavioral health barrier and facilitator factors will be associated with behavioral health treatment engagement.

## Methods

### Participants and procedures

We utilized a subsample of 235 LSMM who were part of an observational longitudinal cohort study (*N* = 290) that focused on engagement in HIV-prevention and behavioral health services [23].

Participants who were enrolled completed an online survey at baseline, 4-months post-baseline, and 8-months post-baseline. The selected subsample (*N* = 235) included LSMM who were classified as having a clinically significant mental health or substance use concern. The subsample of “behavioral health need” was identified based on responses to measures of depression (CES-D-10) [24], anxiety (GAD-7) [25], PTSD (PC-PTSD-5) [26], somatic symptoms (SSS-8) [27], an alcohol use measure (AUDIT-C) [28], and a substance use measure from HPTN083 [29]. See Table 1 for study variable characteristics.

**Table 1:** Descriptive statistics: demographics and key variables (n=235).

Variable	Frequency (%)/ Mean (Standard Deviation)
**Age**	31.26 (8.08)
**Race/Ethnicity**	
White Latino	179 (76.2%)
Black Latino	14 (6%)
Asian Latino	2 (0.9%)
American Indian/Indigenous Latino	11 (4.7%)
Another racial identity and Latino	4 (1.7%)
Multiracial Latino	23 (9.8%)
Declined to answer racial identity and Latino ethnicity	2 (0.9%)
**Nativity**	
Continental United States	114 (48.5%)
Caribbean (e.g., Cuba, Dominican Republic, Puerto Rico)	27 (11.5%)
Central America (e.g., Guatemala, Honduras, Nicaragua, Panama)	16 (6.8%)
North America (Mexico)	2 (0.9%)
South America (e.g., Argentina, Chile, Colombia, Ecuador, Peru, Venezuela, Uruguay, Chile, Brazil, Bolivia)	74 (31.5%)
Born in another country (not listed)	1 (0.4%)
Decline to answer	1 (0.4%)

Full details on the recruitment process can be found elsewhere [23], however, briefly, participants were recruited online, via social media, word of mouth, and a “consent-to-contact” database of previous study participants in South Florida. Participants were screened to verify eligibility which included: (1) identified as a Latino/x or Hispanic gay, bisexual, or man who has sex with men, (2) were aged 18–60, (3) self-report HIV-negative or unknown HIV status, and (4) lived in the greater Miami area in Florida. See S1 File for a diagram depicting the study sample inclusion process. Following verification of the inclusion criteria, participants completed a survey lasting 60–75 minutes and were compensated $40 upon completion of the survey.

#### Statement of ethics.

All study procedures were approved by the University of Miami Institutional Review Board (IRB Protocol Number 20181006). Procedures included approval to conduct this study with a waiver of signed consent, meaning that the investigator obtained consent following the same requirements as written consent, but the participant did not need to sign a consent form. Participants were still provided with consent information within REDCap [30], where the study took place, and indicated their consent to participate by clicking a box prior to survey administration. During the recruitment and consent process, and prior to any data collection, participants were informed that their information would be kept confidential and would only be accessed by study authorized staff members. All data was kept under lock and key (or electronically password protected with access only to authorized research staff). All research staff were trained in ethics, confidentiality, and privacy interest as well as all required trainings for human subjects and behavioral science research, in accordance with University of Miami policies. There was a low likelihood of risk to individuals participating in the proposed study and the potential risk in this study was outweighed by the potential benefits. Participants were able to decline participation or withdraw from the study at any time. The study’s recruitment period was from February 18, 2020 to September 1, 2020 [23].

### Measures

#### Multilevel barriers and facilitators to behavioral health treatment.

Using the information gathered from a qualitative study with LSMM [22], we developed a measure of multilevel barriers and facilitators to behavioral health treatment. Much like Multilevel Barriers and Facilitators to PrEP and Multilevel Barriers and Facilitators to HIV Testing Measures previously developed [31], this behavioral health oriented measure included 49 barrier items and 25 facilitator items. Following procedures utilized for the PrEP and HIV testing measures [31], items were grouped into theoretically derived domains that included ten barrier domains: (1) lack of behavioral health knowledge, (2) lack of perceived need or urgency for behavioral health, (3) behavioral health mistrust and concerns, (4) behavioral health stigma, (5) lack of culturally competent behavioral health providers and outreach, (6) negative behavioral health provider demeanor, (7) clinic and medical system issues for behavioral health, (8) behavioral health privacy concerns, (9) behavioral health cost and insurance issues, and (10) language and immigration concerns and five facilitator domains: (1) behavioral health being normalized, (2) culturally competent behavioral health providers and outreach, (3) behavioral health navigation support, (4) positive behavioral health provider demeanor, and (5) behavioral health affordability. Sample barrier items included, “Not knowing how or where to get behavioral health services” and “My family would think worse of me if I was getting behavioral health services.” Participants rated barriers from 1 (didn’t get in the way of using behavioral health services at all) to 5 (completely got in the way of using behavioral health services). Example facilitator items included, “Someone I trust recommending a specific provider/organization to get behavioral health services” and “Behavioral health services being available for free or low cost.” Participants rated facilitators were from 1 (didn’t or wouldn’t help me get behavioral health services) to 5 (completely did or would help me get behavioral health services).

#### Behavioral health treatment engagement.

We used the Behavioral Health Treatment Cascade as a measure of LSMM’s engagement in behavioral health services [23]. Items that inform LSMM’s position on the cascade are taken from the Psychosocial Treatment Inventory [32] and constructed into a cascade based on a pre-exposure prophylaxis cascade used in the HIV-prevention literature [33]. Based on participants’ responses to the items, they are coded as being in one of the following stages of treatment engagement: (1) pre-contemplation: did not receive behavioral health treatment in past 12 months, no intention to start, (2) contemplation: did not receive treatment in past 12 months, considered starting, (3) preparation: did not receive treatment in past 12 months, planning to start, (4) action and initiation: received at least 1 session of treatment in past 12 months, (5) maintenance and adherence: attended six or more treatment sessions in past 12 months. Higher scores on this measure indicate more behavioral health treatment engagement.

### Data analytic plan

The analytic plan consisted of three steps. First, we examined the hypothesized factor structure of the Multilevel Barriers and Facilitators to Behavioral Health Treatment measure using confirmatory factor analyses (CFA) [34] among the identified sub-sample of 235 LSMM. The hypothesized factor structure consisted of the theoretically derived barrier and facilitator domains described above. Model fit was examined using the Comparative Fit Index (CFI) with values >.90 indicating good fit, Root Mean Square Error of Approximation (RMSEA) and Standardized Root Mean Squared Residual (SRMR) with values <.08 indicating good fit [35,36]. We retained all items with factor loadings > 0.60 [37,38]. We also calculated the composite reliability (coefficient omega) implied by the standardized loadings of each of the resulting factors [39]. Composite reliability is a measure of the internal consistency or reliability of a latent construct in structural equation modeling and assesses the extent to which a set of observed variables (indicators) consistently measures the same underlying latent variable. It is calculated by dividing the sum of the squared factor loadings by the sum of the squared factor loadings plus the sum of the error variances.

Second, we examined discriminant validity by examining the correlations of the identified factors. Third, we examined convergent validity by evaluating the association between identified factors of barriers and facilitators to engagement in behavioral health. Confirmatory factor analyses were conducted in M*P*lus 8.3 [40] and discriminant and convergent validity analyses were conducted in SPSS Version 28 [41]. Missing data in MPlus was handled using full information maximum likelihood (FIML) [42]. Data are available to researchers upon reasonable request to CHARM@miami.edu given that our consent form did not explicitly state that the data would be shared publicly at an individual level.

## Results

### Confirmatory factor analysis

The CFA included seven barrier factors (and therefore, scales): (1) lack of behavioral health knowledge, (2) lack of perceived need or urgency for behavioral health, (3) behavioral health stigma and mistrust, (4) lack of provider skills for working with LSMM, (5) clinic and medical system issues for behavioral health, (6) behavioral health cost and insurance issues, and (7) language and immigration concerns and four facilitator factors (and therefore, scales): (1) peer and provider support and affirmation for seeking behavioral health services, (2) behavioral health navigation support, (3) positive behavioral health provider demeanor, and (4) behavioral affordability. This resulted in 11 first-ordered factors which represent the various domains of barriers and facilitators for behavioral health treatment. Overall, the model fit for the entire sample was acceptable [χ^2^(751) = 1389.28, *p* <.001; RMSEA =.060 (90% CI = [.055,.065]), CFI =.902, SRMR =.055]. With the pre-determined threshold (standardized factor loadings > 0.60), 26 items were excluded from the model. Moreover, based on collinearity between factors and conceptual overlap, three sets of factors were combined: (1) behavioral health is normalized and culturally competent behavioral health providers and outreach, (2) behavioral health mistrust and concerns and behavioral health stigma, and (3) lack of culturally competent behavioral health providers and outreach and negative behavioral health provider demeanor. The final items, composite reliabilities, and standardized factor loadings can be found in Table 2; standardized factor loadings ranged from .652 to .946. Composite reliability implied by the latent factors ranged from .77 to .93.

**Table 2:** Items, composite reliabilities, and factor loadings for behavioral health barriers and facilitators.

Factor	Factor Name	Composite Reliability	Factor Loadings	Item
**Barriers**				
1	Lack of Behavioral Health Knowledge	0.90	0.76	Not knowing that behavioral health services existed
			0.90	Not knowing how or where to get behavioral health services
			0.92	Not knowing enough about behavioral health services to feel comfortable getting this service
2	Lack of Perceived Need or Urgency for Behavioral Health	0.79	0.80	Concerns that behavioral health services aren’t really effective
			0.71	Feeling like it’s better to wait for things to improve/change on their own than get this service
			0.71	Feeling that behavioral health services should only be used as a last resort or in a crisis situation
3	Behavioral Health Stigma and Mistrust^a^	0.90	0.65	Avoiding medications or medical settings in general
			0.74	Concerns about behavioral health services having a negative impact on me
			0.71	Concerns that using behavioral health services could make things worse
			0.81	Being embarrassed about needing behavioral health services
			0.74	Not wanting to talk to a doctor or counselor about my sex life
			0.83	Being uncomfortable asking for behavioral health services
			0.72	Wanting to keep my private life private
4	Lack of Provider Skills for Working with LSMM^b^^,^^c^	0.93	0.88	Behavioral health providers, staff, or organizations not being LGBTQ affirming
			0.83	Behavioral health providers, staff, or organizations not being knowledgeable about Latino/Hispanic people
			0.65	Difficulty finding a provider (counselor, therapist) who is a good fit and understands me
			0.94	The people who provide this service (counselors, therapists) not being caring enough
			0.89	The people who provide this service (counselors, therapists) not being professional enough
5	Clinic and Medical System Issues for Behavioral Health	0.84	0.80	Behavioral health organizations/providers having limited appointments or hours
			0.83	The process for getting behavioral health services taking too long
			0.75	The medical system being confusing or hard to navigate
6	Behavioral Health Cost and Insurance Issues	0.91	0.86	Not having insurance or having insurance that doesn’t cover enough of the cost of behavioral health services
			0.94	Not being able to afford behavioral health services
			0.83	Billing for behavioral health services being a big hassle
7	Language/Immigration Concerns	0.93	0.95	Problems finding behavioral health services in Spanish
			0.92	Problems finding behavioral health services in the same kind of Spanish that I speak (e.g., same country/dialect)
**Facilitators**				
8	Peer and Provider Support and Affirmation for Seeking Behavioral Health Services^d^	0.88	0.77	Seeing/hearing about other Latino men using behavioral health services
			0.70	Seeing/hearing about my friends behavioral health services
			0.81	Behavioral health counselors/ therapists being from the same background as me (Latino, men, gay/bisexual, etc.)
			0.77	The providers, staff, or organization cater to the LGBTQ community
			0.82	The providers, staff, or organization cater to the Latino community
9	Behavioral Health Navigation Support	0.93	0.71	Someone I trust recommending a specific provider/organization to get behavioral health services
			0.78	Someone helping me decide if I should get behavioral health services
			0.80	Someone helping me figure out what to do if I have problems getting the behavioral health services
			0.84	Someone helping me figure out where to go for behavioral health services
			0.89	Someone helping me build up the motivation to get behavioral health services
			0.81	Someone explaining how behavioral health services work
			0.81	Someone holding me accountable and following up to make sure I get behavioral health services
10	Positive Behavioral Health Provider Demeanor	0.85	0.91	Behavioral health counselors/therapists taking a personal and caring approach
			0.81	Behavioral health counselors/therapists being highly professional and business-like
11	Behavioral Health Affordability	0.77	0.72	Having insurance that covers behavioral health services
			0.86	Behavioral health services being available for free or low cost

^a^ Resulting factor based on combining behavioral health mistrust and concerns and behavioral health stigma.

^b^ Resulting factor based on combining lack of culturally competent behavioral health providers and outreach and negative behavioral health provider demeanor.

^c^ LSMM = Latino Sexual Minority Men.

d Resulting factor based on combining behavioral health is normalized and culturally competent behavioral health providers and outreach.

### Discriminant and convergent validity

The majority of the factor correlations ranged from .135 to .754, implying that factors had discriminant validity [43]. Lack of perceived need or urgency for behavioral health correlated strongly with behavioral health stigma and mistrust, however, attempts to combine these factors resulted in poor model fit [χ^2^(774) = 1808.34, *p* <.001; RMSEA =.075 (90% CI = [.071,.080]), CFI =.841, SRMR =.058]. Thus, we retained these two factors as separate factors as simulation studies have shown that correlations between .75 and .90 as not having discriminant validity issues [43]. The correlation between identified barriers and facilitators can be found in S6 File.

Regarding convergent validity, the language and immigration concerns factor (b = -.23, SE = .12, *p* <.05) was significantly associated with less engagement in behavioral health. Conversely, positive behavioral health provider demeanor (*b* =. 37, SE = .13, *p* <.01) was associated with more engagement in behavioral health services. The remaining factors were not significantly associated with behavioral health engagement. S2 File presents sensitivity analysis evaluating means differences in the factors across sample subgroups.

## Discussion

The purpose of this study was to evaluate the factor structure of the Multilevel Barriers and Facilitators to Behavioral Health Treatment and examine the extent to which barriers and facilitators to behavioral health are associated with each other and engagement in behavioral health treatment. Through our factor analysis, we confirmed the following barrier factors: (1) lack of behavioral health knowledge, (2) lack of perceived need or urgency for behavioral health, (3) behavioral health stigma and mistrust, (4) lack of provider skills for working with LSMM, (5) clinic and medical system issues for behavioral health, (6) behavioral health cost and insurance issues, and (7) language and immigration concerns and the following facilitator factors: (1) peer and provider support and affirmation for seeking behavioral health services, (2) behavioral health navigation support, (3) positive behavioral health provider demeanor, and (4) behavioral health affordability, respectively. Additionally, we found evidence of convergent validity for some of these factors, such that language and immigration concerns and positive behavioral health provider demeanor were significantly associated with engagement in behavioral health treatment in the expected directions. Moreover, we found evidence for discriminant validity, suggesting that most of the factors were unrelated to each other. The final and complete Multilevel Barriers and Facilitators to Behavioral Health Measure can be found in English and Spanish in S3 and S4 Files, respectively.

This study established the psychometric properties of the Multilevel Barriers and Facilitators to Behavioral Health Treatment measure, confirming its factor structure, composite reliability, discriminant validity, and convergent validity. This measure captures multilevel determinants of use of behavioral health services among LSMM with a clinically significant mental health or substance use concern, ranging from structural factors related to payment (e.g., financial barriers), to individual factors (e.g., knowledge deficits), that are known to impede engagement with behavioral health services [44]. Among the behavioral health treatment factors identified in this study, the language and immigration concerns factor was negatively associated with engagement in behavioral health services. Overall, Latinos are less likely to engage in behavioral health treatment than non-Latino whites [45], contributing to the existing disparities in behavioral health treatment. These disparities are further exacerbated among immigrant LSMM due, in part, to concerns that engagement in treatment may negatively impact their immigration status, particularly among undocumented LSMM [46]. In addition to providing evidence of convergent validity, the negative association between the language and immigration concerns factor and behavioral health engagement implies that LSMM likely experience compounded difficulty engaging in behavioral health services relative to heterosexual, non-Latino, white individuals [15,17].

We also found that positive behavioral health provider demeanor was positively associated with engagement in behavioral health treatment. A study by Alegría et al. [47] found that Latino patients’ preferences for behavioral health treatment were related to ensuring that providers were clearly paying attention to what the patient was saying. Moreover, the extant literature has shown that providers with a positive demeanor (e.g., taking a personal and caring approach) are critical for promoting LSMM’s engagement in behavioral health services [48–50]. A final finding in our study was related to discriminant validity. We found that the majority of factors showed discriminant validity, suggesting that the factors were conceptually unrelated to each other. An exception was the correlation between lack of perceived need or urgency for behavioral health and behavioral health stigma and mistrust, which showed a strong correlation. It may be that the two factors are conceptually similar such that stigma and mistrust regarding behavioral health subsequently drives a lack of perceived need or urgency for behavioral health among LSMM. Efforts to combine the two factors resulted in poor model fit, thus the factors were retained as separate.

The Multilevel Barriers and Facilitators to Behavioral Health Measure is a tool for behavioral health researchers to quantitatively assess multilevel determinants that exist at multiple levels of LSMMs’ contexts. Moreover, the tool’s availability in English and Spanish (see S3 and S4 Files) ensures that it is accessible to a broad audience, thus facilitating cross-cultural comparisons and integration into studies with diverse participant pools. This multilevel understanding of barriers *and* facilitators unique to LSMM (e.g., factors related to immigration and language) can be captured and used to inform mental health services researchers and policymakers with ways to engage LSMM with a clinically significant mental health or substance use concern in behavioral health treatment. For instance, from distal to proximal levels, the measure captures structural factors (e.g., insurance issues), interpersonal factors (e.g., lack of provider skills for working with LSMM), and individual factors (e.g., lack of behavioral health knowledge). Moreover, this measure can provide mental health services researchers and policymakers with insight as to how certain barriers and/or facilitators are relevant to specific groups. This information can subsequently inform culturally tailored approaches to addressing behavioral health treatment disparities affecting LSMM with a clinically significant mental health or substance use concern. In addition to providing insight into culturally tailored approaches to addressing behavioral health treatment disparities, the measure can be used to compare service providers and assess variations in practice based on responses to provider skills for working with LSMM. Such data would provide a benchmark to rank providers—from best to worst—based on their effectiveness in mitigating barriers and enhancing facilitators, thereby identifying best practices to engage LSMM in behavioral health treatment.

Our analysis also points to structural barriers (i.e., language and immigration) specifically relevant to LSMM that hinder their engagement in behavioral health treatment and interpersonal factors (i.e., positive behavioral health provider demeanor) that can enable engagement in behavioral health treatment. This study’s findings suggest addressing the identified factors requires a multi-pronged, culturally congruent strategy which includes building trust and ensuring safety to assuage immigration and language related concerns and training behavioral health practitioners to take a personal and caring approach to treating their patients.

This study should be interpreted while considering the following limitations. Similar to other studies evaluating barriers and facilitators of uptake of services, one common limitation is that this study was a cross-sectional study, and barriers and facilitators may vary as a function of time [31,51]. Future studies should examine these determinants longitudinally and evaluate how barrier and facilitators to behavioral health treatment change over time. For example, as LSMM become engaged in behavioral health treatment, it is likely that certain barriers and facilitators may no longer be relevant. Among our sample, 213 (91%) were available for future follow-ups at four months post-baseline and 201 (86%) were available for future follow-ups at eight months post-baseline thus permitting future research evaluating the trajectories of barrier and facilitators to behavioral health treatment over time. Second, this study was conducted in one geographical region, South Florida, and may therefore not be generalizable to other samples of LSMM in other geographic areas [31,51]. Third, there is a possible self-reporting bias of questions – we analyzed data using a subgroup of LSMM who had a clinically significant mental health or substance use concern. It may be that there were other participants who underreported questions related to mental health or substance use that were not captured in the present sample. Fourth, our sample was *N* = 235, however, researchers recommend a minimum sample size of around 200 for CFA [52,53] and all items showed high factor loadings (ranging from .652 to .946) and strong composite reliabilities ranging from .77 to .93. Future studies should evaluate the measure in larger samples and pursue measurement invariance analysis to identify possible variability across specific subgroups. As an example, incorporating this bilingual measure into large national studies that include LSMM (e.g., All of Us, Pride Study) enables researchers to compare behavioral health service experiences across diverse populations and regions, and to understand how systemic factors influence treatment engagement to replicate, extend, and provide more evidence on the measure. Finally, two of the identified factors (lack of perceived need or urgency for behavioral health and behavioral health mistrust and stigma) did not show discriminant validity. Despite this, all factors showed strong composite reliabilities and factor loadings, suggesting that the resultant factor structure has appropriate psychometric properties.

### Conclusions and recommendations

In conclusion, we developed a measure of barriers and facilitators to behavioral health treatment that considers multiple levels of LSMMs’ contexts and is culturally syntonic. Moreover, we evaluated the association of the measure with engagement with behavioral health treatment and provided evidence of this measure’s validity. We recommend use of this measure for mental health and substance use services researchers and policymakers regarding how to improve the reach of behavioral health treatment services to LSMM, and in turn, reduce behavioral health disparities among this population.

## Supporting information

S1 File STROBE flow chart. (DOCX)

S2 File Sensitivity analysis. (DOCX)

S3 File English measure. (DOCX)

S4 File Spanish measure. (DOCX)

S5 File STROBE checklist. (DOCX)

S6 File Correlations between the identified barrier and facilitator factors. (DOCX)
