# Supplementary material for: Multilevel barriers and facilitators to behavioral health treatment among Latino sexual minority men
Source: PLOS Ment Health. 2025 Apr 21;2(4):e0000153. doi: 10.1371/journal.pmen.0000153 (PMC12798582; doi:10.1371/journal.pmen.0000153)
Supplement: S1 File — (DOCX) [file pmen.0000153.s001.docx]

STROBE flow chart. STROBE, Strengthening the Reporting of Observational Studies in Epidemiology.

Assessed for eligibility by September 1, 2020 (n=302)

Included (n=290)

Excluded (n=12)

-Invalid* or duplicate (n=12)
*Surveys were considered invalid if participant failed attention check questions

Analyzed (n=235)

Excluded (n=55)

-Did not have a clinically significant mental health or substance use concern (n=55)

Inclusion

Analysis

Identification
