# Supplementary material for: Multilevel barriers and facilitators to behavioral health treatment among Latino sexual minority men
Source: PLOS Ment Health. 2025 Apr 21;2(4):e0000153. doi: 10.1371/journal.pmen.0000153 (PMC12798582; doi:10.1371/journal.pmen.0000153)
Supplement: S2 File — (DOCX) [file pmen.0000153.s002.docx]

**S2 File.** Sensitivity Analysis

| **Barriers** | **Mental Health Problem Only** | **Substance Use Problem Only** | **Mental Health and Substance Use Problem** |
| --- | --- | --- | --- |
| Lack of Behavioral Health Knowledge* | 2.50 (1.36) | 1.94 (1.22) | 2.64 (1.42) |
| Lack of Perceived Need or Urgency for Behavioral Health* | 2.22 (1.19) | 1.73 (0.93) | 2.33 (1.19) |
| Behavioral Health Stigma and Mistrust*** | 2.17 (1.13) | 1.56 (0.73) | 2.42 (1.17) |
| Lack of Provider Skills for Working with LSMM*** | 2.24 (1.23) | 1.71 (0.96) | 2.62 (1.31) |
| Clinic and Medical System Issues for Behavioral Health*** | 2.48 (1.29) | 1.67 (0.98) | 2.60 (1.28) |
| Behavioral Health Cost and Insurance Issues** | 3.06 (1.57) | 2.20 (1.43) | 3.14 (1.53) |
| Language and Immigration Concerns* | 1.44 (1.03) | 1.06 (0.26) | 1.51 (1.10) |
| **Facilitators** |  |  |  |
| Peer and Provider Support and Affirmation for Seeking Behavioral Health Services | 3.45 (1.30) | 3.27 (1.29) | 3.71 (1.18) |
| Behavioral Health Navigation Support | 3.61 (1.27) | 3.31 (1.30) | 3.70 (1.18) |
| Positive Behavioral Health Provider Demeanor | 3.86 (1.32) | 3.73 (1.30) | 4.16 (1.08) |
| Behavioral Health Affordability | 4.21 (1.30) | 4.29 (1.14) | 4.23 (1.15) |

**(a) Mental Health and/or Substance Use**

**p* < .05, ***p* < .01, ****p* < .001

There were significant differences across subgroups such that LSMM with mental health and substance use problems had significantly higher means across all factors relative to those with substance use problems only. LSMM with mental health problems only had significantly higher means of behavioral health stigma and mistrust, lack of provider skills for working with LSMM, clinic and medical system issues for behavioral health, and behavioral health cost and insurance issues, relative to LSMM with substance use problems only.

**(b) Language Preference**

| **Barriers** | **English** | **Spanish** | **Both** |
| --- | --- | --- | --- |
| Lack of Behavioral Health Knowledge | 2.36 (1.32) | 2.43 (1.35) | 2.65 (1.51) |
| Lack of Perceived Need or Urgency for Behavioral Health | 2.18 (1.14) | 2.18 (1.21) | 2.17 (1.20) |
| Behavioral Health Stigma and Mistrust | 2.17 (1.16) | 2.16 (1.05) | 2.10 (1.10) |
| Lack of Provider Skills for Working with LSMM | 2.31 (1.28) | 2.09 (1.26) | 2.34 (1.20) |
| Clinic and Medical System Issues for Behavioral Health | 2.37 (1.29) | 2.37 (1.24) | 2.43 (1.28) |
| Behavioral Health Cost and Insurance Issues | 2.85 (1.56) | 2.88 (1.52) | 3.15 (1.60) |
| Language and Immigration Concerns*** | 1.22 (0.73) | 2.03 (1.37) | 1.46 (1.08) |
| **Facilitators** |  |  |  |
| Peer and Provider Support and Affirmation for Seeking Behavioral Health Services | 3.39 (1.27) | 3.64 (1.40) | 3.69 (1.16) |
| Behavioral Health Navigation Support | 3.54 (1.27) | 3.48 (1.31) | 3.76 (1.18) |
| Positive Behavioral Health Provider Demeanor | 3.89 (1.28) | 3.76 (1.38) | 4.13 (1.08) |
| Behavioral Health Affordability | 4.14 (1.30) | 4.12 (1.29) | 4.49 (0.93) |

**p* < .05, ***p* < .01, ****p* < .001

There were significant differences across subgroups such that LSMM who preferred speaking Spanish, had significantly higher language and immigration concerns relative to those who preferred English or both English and Spanish.
