# Supplementary material for: Multilevel barriers and facilitators to behavioral health treatment among Latino sexual minority men
Source: PLOS Ment Health. 2025 Apr 21;2(4):e0000153. doi: 10.1371/journal.pmen.0000153 (PMC12798582; doi:10.1371/journal.pmen.0000153)
Supplement: S3 File — (DOCX) [file pmen.0000153.s003.docx]

**Multilevel Barriers and Facilitators to Behavioral Health Treatment Measure**

**Barriers**

“For each factor listed below, please answer if it "Didn't get in the way of using behavioral health services at all," "got in the way a little bit," "somewhat got in the way," "very much got in the way," or "completely got in the way" of using behavioral health services.”

| **Barriers** | **Items** | **Didn’t get in the way of using behavioral health services at all** | **Got in the way a little bit** | **Somewhat got in the way** | **Very much got in the way** | **Completely got in the way of using behavioral health services** |
| --- | --- | --- | --- | --- | --- | --- |
| Lack of Behavioral Health Knowledge | Not knowing that behavioral health services existed | 1 | 2 | 3 | 4 | 5 |
|  | Not knowing how or where to get behavioral health services | 1 | 2 | 3 | 4 | 5 |
|  | Not knowing enough about behavioral health services to feel comfortable getting this service | 1 | 2 | 3 | 4 | 5 |
| Lack of Perceived Need or Urgency for Behavioral Health | Concerns that behavioral health services aren't really effective | 1 | 2 | 3 | 4 | 5 |
|  | Feeling like it's better to wait for things to improve/change on their own than get this service | 1 | 2 | 3 | 4 | 5 |
|  | Feeling that behavioral health services should only be used as a last resort or in a crisis situation | 1 | 2 | 3 | 4 | 5 |
| Behavioral Health Stigma and Mistrust | Avoiding medications or medical settings in general | 1 | 2 | 3 | 4 | 5 |
|  | Concerns about behavioral health services having a negative impact on me | 1 | 2 | 3 | 4 | 5 |
|  | Concerns that using behavioral health services could make things worse | 1 | 2 | 3 | 4 | 5 |
|  | Being embarrassed about needing behavioral health services | 1 | 2 | 3 | 4 | 5 |
|  | Not wanting to talk to a doctor or counselor about my sex life | 1 | 2 | 3 | 4 | 5 |
|  | Being uncomfortable asking for behavioral health services | 1 | 2 | 3 | 4 | 5 |
|  | Wanting to keep my private life private | 1 | 2 | 3 | 4 | 5 |
| Lack of Provider Skills for Working with LSMM | Behavioral health providers, staff, or organizations not being LGBTQ affirming | 1 | 2 | 3 | 4 | 5 |
|  | Behavioral health providers, staff, or organizations not being knowledgeable about Latino/Hispanic people | 1 | 2 | 3 | 4 | 5 |
|  | Difficulty finding a provider (counselor, therapist) who is a good fit and understands me | 1 | 2 | 3 | 4 | 5 |
|  | The people who provide this service (counselors, therapists) not being caring enough | 1 | 2 | 3 | 4 | 5 |
|  | The people who provide this service (counselors, therapists) not being professional enough | 1 | 2 | 3 | 4 | 5 |
| Clinic and Medical System Issues for Behavioral Health | Behavioral health organizations/providers having limited appointments or hours | 1 | 2 | 3 | 4 | 5 |
|  | The process for getting behavioral health services taking too long | 1 | 2 | 3 | 4 | 5 |
|  | The medical system being confusing or hard to navigate | 1 | 2 | 3 | 4 | 5 |
| Behavioral Health Cost and Insurance Issues | Not having insurance or having insurance that doesn't cover enough of the cost of behavioral health services | 1 | 2 | 3 | 4 | 5 |
|  | Not being able to afford behavioral health services | 1 | 2 | 3 | 4 | 5 |
|  | Billing for behavioral health services being a big hassle | 1 | 2 | 3 | 4 | 5 |
| Language and Immigration Concerns | Problems finding behavioral health services in Spanish | 1 | 2 | 3 | 4 | 5 |
|  | Problems finding behavioral health services in the same kind of Spanish that I speak (e.g., same country/dialect) | 1 | 2 | 3 | 4 | 5 |

**Facilitators**

“For each factor listed below, please answer if it "didn't/wouldn't help you get behavioral health services at all," "helped a little bit," "helped you somewhat," "helped you very much," or "completely did/would help you get behavioral health services."”

| **Facilitators** | **Items** | **Didn’t or wouldn’t help me get behavioral health services** | **Helped a little bit** | **Helped you somewhat** | **Helped you very much** | **Completely did or would help me get behavioral health services** |
| --- | --- | --- | --- | --- | --- | --- |
| Peer and Provider Support and Affirmation for Seeking Behavioral Health Services | Seeing/hearing about other Latino men using behavioral health service | 1 | 2 | 3 | 4 | 5 |
|  | Seeing/hearing about my friends behavioral health services | 1 | 2 | 3 | 4 | 5 |
|  | Behavioral health counselors/ therapists being from the same background as me (Latino, men, gay/bisexual, etc.) | 1 | 2 | 3 | 4 | 5 |
|  | A friend suggesting that I use PrEP | 1 | 2 | 3 | 4 | 5 |
|  | The providers, staff, or organization cater to the LGBTQ community | 1 | 2 | 3 | 4 | 5 |
|  | The providers, staff, or organization cater to the Latino community | 1 | 2 | 3 | 4 | 5 |
| Behavioral Health Navigation Support | Someone I trust recommending a specific provider/organization to get behavioral health services | 1 | 2 | 3 | 4 | 5 |
|  | Someone helping me decide if I should get behavioral health services | 1 | 2 | 3 | 4 | 5 |
|  | Someone helping me figure out what to do if I have problems getting the behavioral health services | 1 | 2 | 3 | 4 | 5 |
|  | Someone helping me figure out where to go for behavioral health services | 1 | 2 | 3 | 4 | 5 |
|  | Someone helping me build up the motivation to get behavioral health services | 1 | 2 | 3 | 4 | 5 |
|  | Someone explaining how behavioral health services work | 1 | 2 | 3 | 4 | 5 |
|  | Someone holding me accountable and following up to make sure I get behavioral health services | 1 | 2 | 3 | 4 | 5 |
| Positive Behavioral Health Provider Demeanor | Behavioral health counselors/therapists taking a personal and caring approach | 1 | 2 | 3 | 4 | 5 |
|  | Behavioral health counselors/therapists being highly professional and business-like | 1 | 2 | 3 | 4 | 5 |
| Behavioral Health Affordability | Having insurance that covers behavioral health services | 1 | 2 | 3 | 4 | 5 |
|  | Behavioral health services being available for free or low cost | 1 | 2 | 3 | 4 | 5 |

**Scoring Instructions:**

This measure does not produce an overall score. Rather, a mean score is calculated for each cluster of items above. For example, a person’s “Lack of Behavioral Health Knowledge” scale score would be calculated by taking the mean of the three items within that scale. Scores range from 1 – 5. For the barriers, higher scores indicate that domain was more of a barrier to behavioral health treatment for the individual completing the measure. For facilitators, higher scores indicate that domain was or would be more of a facilitator to behavioral health treatment for the individual completing the measure. The Multilevel Barriers and Facilitators to Behavioral Health Treatment Measure can be administered in its entirety, or selected items from each cluster (e.g., Behavioral Health Affordability) can be administered.
