# Supplementary material for: Multilevel barriers and facilitators to behavioral health treatment among Latino sexual minority men
Source: PLOS Ment Health. 2025 Apr 21;2(4):e0000153. doi: 10.1371/journal.pmen.0000153 (PMC12798582; doi:10.1371/journal.pmen.0000153)
Supplement: S4 File — (DOCX) [file pmen.0000153.s004.docx]

**Multilevel Barriers and Facilitators to PrEP Measure (Spanish)**

**Barreras**

“Para cada pregunta a continuación, por favor responda si fue “Nada de un obstáculo para usar los servicios de salud conductual,” “Un poco de un obstáculo,” “Algo de un obstáculo,” “Mucho de un obstáculo,” o “Un obstáculo totalmente para usar los servicios de salud conductual.””

| **Barriers** | **Items** | **Nada de un obstáculo para usar los servicios de salud conductual** | **Un poco de un obstáculo** | **Algo de un obstáculo** | **Mucho de un obstáculo** | **Un obstáculo totalmente para usar los servicios de salud conductual** |
| --- | --- | --- | --- | --- | --- | --- |
| Lack of Behavioral Health Knowledge | No sabiendo que los servicios de salud conductual existían | 1 | 2 | 3 | 4 | 5 |
|  | No sabiendo cómo o dónde obtener los servicios de salud conductual | 1 | 2 | 3 | 4 | 5 |
|  | No sabiendo lo suficiente de los servicios de salud conductual para sentirse cómodo usándolos | 1 | 2 | 3 | 4 | 5 |
| Lack of Perceived Need or Urgency for Behavioral Health | Preocupaciones de que los servicios de salud conductual no son efectivos | 1 | 2 | 3 | 4 | 5 |
|  | Sentir que es mejor esperar para que cosas mejoren/cambien en vez de usar los servicios de salud conductual | 1 | 2 | 3 | 4 | 5 |
|  | Sintiendo que los servicios de salud conductual solo se deberían usar como último recurso o en emergencias | 1 | 2 | 3 | 4 | 5 |
| Behavioral Health Stigma and Mistrust | Evitando medicamentos o entornos médicos en general | 1 | 2 | 3 | 4 | 5 |
|  | Preocupaciones sobre los servicios de salud conductual teniendo un impacto negativo en mí | 1 | 2 | 3 | 4 | 5 |
|  | Preocupaciones que el uso de los servicios de salud conductual podrían empeorar cosas | 1 | 2 | 3 | 4 | 5 |
|  | Estando avergonzado de necesitar servicios de salud conductual | 1 | 2 | 3 | 4 | 5 |
|  | No queriendo hablar con un doctor o un proveedor de salud sobre mi vida sexual | 1 | 2 | 3 | 4 | 5 |
|  | Estando incomodo solicitando servicios de salud conductual | 1 | 2 | 3 | 4 | 5 |
|  | Queriendo mantener mi vida privada en privado | 1 | 2 | 3 | 4 | 5 |
| Lack of Provider Skills for Working with LSMM | Los proveedores, los empleados o la organización ofreciendo servicios de salud conductual no siendo amigable con la comunidad LGBTQ. | 1 | 2 | 3 | 4 | 5 |
|  | Los proveedores, los empleados o la organización ofreciendo servicios de salud conductual no siendo informados sobre la comunidad latino/hispano | 1 | 2 | 3 | 4 | 5 |
|  | Dificultad encontrando un proveedor para servicios de salud conductual quien sería bueno y me entendería | 1 | 2 | 3 | 4 | 5 |
|  | La gente que ofrecen los servicios de salud conductual (consejeros, terapeutas) no siendo lo suficiente cariñoso | 1 | 2 | 3 | 4 | 5 |
|  | La gente que ofrecen los servicios de salud conductual (consejeros, terapeutas) no siendo lo suficiente profesional | 1 | 2 | 3 | 4 | 5 |
| Clinic and Medical System Issues for Behavioral Health | Organizaciones teniendo citas o horarios limitados | 1 | 2 | 3 | 4 | 5 |
|  | El proceso para recibir servicios de salud conductual es demasiado largo | 1 | 2 | 3 | 4 | 5 |
|  | El sistema médico siendo confuso o difícil de navegar | 1 | 2 | 3 | 4 | 5 |
| Behavioral Health Cost and Insurance Issues | No teniendo seguro médico que cubre lo suficiente del costo de recibir servicios de salud conductual | 1 | 2 | 3 | 4 | 5 |
|  | Pensando que no podría pagar por los servicios de salud conductual. | 1 | 2 | 3 | 4 | 5 |
|  | La facturación para servicios de salud conductual es una gran molestia | 1 | 2 | 3 | 4 | 5 |
| Language and Immigration Concerns | Problemas encontrando servicios de salud conductual en español | 1 | 2 | 3 | 4 | 5 |
|  | Problemas encontrando servicios de salud conductual en el mismo *tipo* de español que hablo (p. ej., mismo país/dialecto) | 1 | 2 | 3 | 4 | 5 |

**Facilitadores**

“Para cada factor enumerado a continuación, por favor responda si “ayudaría / ayudó a obtener los servicios de salud conductual,” le “ayudó un poco,” le “ayudó,” le “ayudó mucho,” o le “ayudó completamente/me ayudaría a obtener los servicios de salud conductual.””

| **Facilitators** | **Items** | **No me ayudaría / no me ayudó a obtener los servicios de salud conductual** | **Ayudó un poco** | **Ayudó** | **Ayudó mucho** | **Me ayudó completamente/me ayudaría a obtener los servicios de salud conductual** |
| --- | --- | --- | --- | --- | --- | --- |
| Peer and Provider Support and Affirmation for Seeking Behavioral Health Services | Viendo/escuchando sobre otros hombres Latinos que usan servicios de salud conductual | 1 | 2 | 3 | 4 | 5 |
|  | Viendo/escuchando que mis amigos usan servicios de salud conductual | 1 | 2 | 3 | 4 | 5 |
|  | Los proveedores/empleados de servicios de salud conductual siendo de la mismo identidad que mí (Latino, hombre, gay/bisexual, etc.) | 1 | 2 | 3 | 4 | 5 |
|  | Los proveedores, empleados, o la organización ofreciendo servicios de salud conductual atienden la comunidad LGBTQ | 1 | 2 | 3 | 4 | 5 |
|  | Los proveedores/empleados de servicios de salud conductual atienden la comunidad Latino | 1 | 2 | 3 | 4 | 5 |
| Behavioral Health Navigation Support | Alguien quien confió recomendándome un proveedor/una organización específica para obtener servicios de salud conductual | 1 | 2 | 3 | 4 | 5 |
|  | Alguien ayudándome decidir si debería usar servicios de salud conductual | 1 | 2 | 3 | 4 | 5 |
|  | Alguien ayudándome solucionar que hacer si tengo problemas obteniendo servicios de salud conductual | 1 | 2 | 3 | 4 | 5 |
|  | Alguien ayudándome solucionare dónde ir para obtener servicios de salud conductual | 1 | 2 | 3 | 4 | 5 |
|  | Alguien ayudándome desarrollar la motivación para obtener servicios de salud conductual | 1 | 2 | 3 | 4 | 5 |
|  | Alguien explicándome como trabajan los servicios de salud conductual | 1 | 2 | 3 | 4 | 5 |
|  | Alguien haciéndome responsable y haciéndose un seguimiento para asegurase que obtenga servicios de salud conductual | 1 | 2 | 3 | 4 | 5 |
| Positive Behavioral Health Provider Demeanor | Consejeros/terapeutas de los servicios de salud conductual usando enfoque personalizado y cariñoso | 1 | 2 | 3 | 4 | 5 |
|  | Consejeros/terapeutas de los servicios de salud conductual siendo altamente profesionales y formales | 1 | 2 | 3 | 4 | 5 |
| Behavioral Health Affordability | Teniendo seguro médico que cubre los servicios de salud conductual | 1 | 2 | 3 | 4 | 5 |
|  | Servicios de salud conductual siendo disponible de forma gratuita o bajo costo | 1 | 2 | 3 | 4 | 5 |

**Scoring Instructions:**

This measure does not produce an overall score. Rather, a mean score is calculated for each cluster of items above. For example, a person’s “Lack of Behavioral Health Knowledge” scale score would be calculated by taking the mean of the three items within that scale. Scores range from 1 – 5. For the barriers, higher scores indicate that domain was more of a barrier to behavioral health treatment for the individual completing the measure. For facilitators, higher scores indicate that domain was or would be more of a facilitator to behavioral health treatment for the individual completing the measure. The Multilevel Barriers and Facilitators to Behavioral Health Treatment Measure can be administered in its entirety, or selected items from each cluster (e.g., Behavioral Health Affordability) can be administered.
