# Supplementary material for: Multilevel barriers and facilitators to behavioral health treatment among Latino sexual minority men
Source: PLOS Ment Health. 2025 Apr 21;2(4):e0000153. doi: 10.1371/journal.pmen.0000153 (PMC12798582; doi:10.1371/journal.pmen.0000153)
Supplement: S6 File — (DOCX) [file pmen.0000153.s006.docx]

| Factor | 1 | 2 | 3 | 4 | 5 | 6 | 7 | 8 | 9 | 10 | 11 |
| --- | --- | --- | --- | --- | --- | --- | --- | --- | --- | --- | --- |
| 1. Lack of Behavioral Health Knowledge | - |  |  |  |  |  |  |  |  |  |  |
| 1. Lack of Perceived Need or Urgency for Behavioral Health | 0.51*** | - |  |  |  |  |  |  |  |  |  |
| 1. Behavioral Health Stigma and Mistrust | 0.60*** | 0.88*** | - |  |  |  |  |  |  |  |  |
| 1. Lack Of Provider Skills for Working With LSMM^a^ | 0.46*** | 0.71*** | 0.66*** | - |  |  |  |  |  |  |  |
| 1. Clinic and Medical System Issues for Behavioral Health | 0.56*** | 0.75*** | 0.75*** | 0.70*** | - |  |  |  |  |  |  |
| 1. Behavioral Health Cost and Insurance Issues | 0.40*** | 0.41*** | 0.34*** | 0.49*** | 0.61*** | - |  |  |  |  |  |
| 1. Language/Immigration Concerns | 0.25** | 0.51*** | 0.37*** | 0.42*** | 0.46*** | 0.28*** | - |  |  |  |  |
| 1. Peer And Provider Support and Affirmation for Seeking Behavioral Health Services | 0.23** | 0.21** | 0.23** | 0.33*** | 0.22** | 0.18* | 0.25*** | - |  |  |  |
| 1. Behavioral Health Navigation Support | 0.32*** | 0.25** | 0.24** | 0.25*** | 0.30*** | 0.21** | 0.15** | 0.74*** | - |  |  |
| 1. Positive Behavioral Health Provider Demeanor | 0.14 | 0.24** | 0.21** | 0.31*** | 0.21** | 0.22** | 0.15** | 0.79*** | 0.74*** | - |  |
| 1. Behavioral Health Affordability | 0.19* | 0.26*** | 0.18* | 0.21** | 0.34*** | 0.347*** | 0.12** | 0.57*** | 0.72*** | 0.70*** | - |

**S6 File. Correlations Between the Identified Barrier and Facilitator Factors**

**p* < 0.05, ***p* < 0.01, ****p* < 0.001

^a^LSMM = Latino Sexual Minority Men.
